# Effects of person-job fit on occupational commitment among kindergarten teachers: occupational well-being as mediator and perceived organizational support as moderator

**DOI:** 10.1186/s40359-023-01441-7

**Published:** 2023-11-20

**Authors:** Weiwei Huang, Shuyue Zhang, Hui Li

**Affiliations:** 1 School of Education, Zhaoqing University, Zhaoqing, China; 2 Faculty of Education, Guangxi Normal University, Guilin, China

**Keywords:** Kindergarten teachers, Occupational commitment, Person-job fit, Occupational well-being, Perceived organizational support

## Abstract

**Introduction:**

The objective of this research was to investigate the effect of person-job fit on occupational commitment among Chinese kindergarten teachers, and to identify strategies for improving the occupational commitment of this professional group.

**Methods:**

A survey was conducted among kindergarten teachers utilizing the Occupational Commitment Scale, Person-job Fit Scale, Occupational Well-being Scale, and Perceived Organizational Support Scale, resulting in the acquisition of 1539 valid data.

**Results:**

A significant positive correlation was observed between person-job fit and occupational commitment, with occupational well-being serving as a partial mediator in this association. Additionally, the direct effect of person-job fit on occupational commitment was moderated by perceived organizational support. Specifically, a significant positive correlation between person-job fit and occupational commitment was evident when the scores of perceived organizational support were below 0.21, whereas a significant negative correlation was observed when the scores of perceived organizational support were above 1.67.

**Conclusions:**

In order to enhance the degree of occupational commitment among kindergarten teachers, it is imperative to pay attention to their person-job fit, perceived organizational support, and occupational well-being.

## Introduction

Kindergarten teachers hold a crucial position in fostering the overall development of children, serving as the primary facilitators of direct educational endeavors. Nevertheless, the turnover rate of kindergarten teachers remains considerably elevated, particularly within East Asian societies [1]. The COVID-19 pandemic has had a detrimental effect on the remuneration of these educators, leading to a notable surge in resignations and career changes within the kindergarten teaching sector [2]. Within four months after the outbreak of the epidemic, the turnover rate of kindergarten teachers in China was 19.4% [3], far exceeding the annual turnover rate of teachers in Finland, Singapore and other places of about 4%, and also exceeding the annual turnover rate of preschool and early childhood care teachers in the United States of 15% [4]. The high turnover rate of kindergarten teachers in China has hindered the smooth implementation of various kindergarten work during the epidemic period, which has aroused the concern of the education community about the low stability of kindergarten teachers [2, 3, 5]. The considerable rate of turnover observed among kindergarten teachers serves as a crucial obstacle to the progress of early childhood education in China, as indicated by recent research [5–7]. High turnover of kindergarten teachers poses a significant threat to the well-being of young children, as frequent classroom changes of teachers tend to reduce social interaction and reduce the procedural quality of kindergarten education [8]. Consequently, tackling this matter and formulating strategies to enhance teacher retention has emerged as an urgent concern for both Chinese preschool education administrators and scholars [7].

The provision of preschool education in China is divided into kindergarten education catering to children aged 3–6 and childcare services catering to children below 3. As of 2020, the gross enrollment rate for kindergarten education across China has attained 85.2%. Presently, the focus of China’s kindergarten education is transitioning from a developmental scale to a developmental quality stage. The quality of preschool education relies on the size and stability of the preschool teaching workforce, as indicated by Liu and Song [7]. The attrition of teachers poses a challenge for kindergartens to uphold their initial teaching standards, thereby diminishing the core competitiveness of these institutions. Gao and Li have identified the primary goals for stabilizing the kindergarten teacher workforce, which encompass minimizing turnover rates and bolstering retention rates [9]. However, existing scholarly literature has primarily focused on reducing turnover rates among kindergarten teachers [5, 10], with a scarcity of research from the perspective of retention. Based on the principles of positive psychology [11], it is crucial to ascertain positive orientations and explore strategies to attain them, rather than exclusively concentrating on avoiding negative objectives. As a result, it holds significant practical value to investigate the factors that promote teacher retention in the kindergarten setting.

According to the research, the significance of teachers’ occupational commitment extends beyond mitigating the prevalent issue of teacher turnover rates in the global education industry [12], as it also plays a crucial role in enhancing educator retention [13]. According to Meyer et al., the decision of employees to maintain or terminate their professional membership is influenced by their occupational commitment, which can be classified into affective commitment (stemming from strong emotional ties), continuance commitment (stemming from the high cost of changing employers or careers), and normative commitment (stemming from a sense of moral responsibility) [14]. Yüksek et al. further posit that occupational commitment functions as an intrinsic motivator that unconsciously directs individuals’ actions [15]. According to Collie, the affective commitment of teachers is indicative of their sense of affiliation with their profession [16]. In the case of kindergarten teachers, occupational commitment is a crucial aspect of their professional growth and serves as a significant predictor of their sustained engagement and dedication to the field [17]. As noted by Lee et al., heightened levels of occupational commitment among kindergarten teachers can facilitate their active contribution towards enhancing the quality of early childhood education and care [18]. However, when COVID-19 occurred, the high turnover rate of kindergarten teachers not only affected the development of various work areas of kindergarten education, but also made all sectors of society clearly see the problem of low occupational commitment of kindergarten teachers in China like a mirror. So, the objective of this study is to identify the factors that influence the occupational commitment of kindergarten teachers and explore strategies to enhance their commitment, ultimately leading to improved teacher retention rates.

Given the need for individualized care and education for young students, kindergarten teachers must possess comprehensive and flexible competencies, making the alignment between teachers and their positions a crucial aspect to consider [19]. As the push to improve the quality of kindergarten education gains traction, it is crucial to attract early childhood education professionals who are in line with the organizational culture and developmental philosophy of kindergartens, and who possess a strong commitment to kindergarten education. Kindergarten teachers who exhibit a high level of person-job fit can effectively fulfill their duties in educating children with excellence, thereby enhancing the overall quality of education [10]. According to Yang et al., individuals who exhibit a strong person-job fit experience a heightened sense of job control, which can mitigate the adverse effect of job stress on occupational commitment [20]. Therefore, person-job fit may serve as a safeguarding element for occupational commitment.

The person-job fit theory posits that aligning an individual’s occupational ideals, values, and preferred reinforcement with their jobs can result in positive evaluations and emotional experiences of the work environment, thereby promoting continued engagement in the current job [21]. This implies that occupational well-being may serve as a mediator between person-job fit and occupational commitment. Additionally, the protective-protective model suggests that an individual’s adaptation outcome is contingent upon the interplay of two protective factors [22]. In essence, the effect of a single protective factor on an individual’s adaptation outcome, such as occupational commitment, may be subject to the influence of another protective factor. Social support, as a significant social variable, exerts an effect on employees’ occupational commitment, thereby enhancing their ability to effectively navigate the occupational milieu they encounter [23]. Based on the job demand-resources (JD-R) theory, job resources are known to enhance motivation and foster stronger commitment [24]. Perceived organizational support is not only an important social support, but also a crucial job resource, suggesting that perceived organizational support is an additional protective factor for occupational commitment. Consequently, perceived organizational support was employed as a moderating variable in this study.

## Literature review

### Kindergarten teachers in China

The total number of kindergarten principals and full-time teachers in China will exceed 3.5 million [25]. Although the Chinese government has placed increasing emphasis on preschool education, it has not been integrated into the formal school system as in Sweden and other countries [26], and kindergarten teachers do not command the same respect as primary and secondary school teachers. Consequently, preschool education in China remains marginalized. Most kindergarten teachers in China are employed on a temporary basis and receive low wages. In certain kindergartens, the percentage of temporary teachers exceeds 80% [27]. A study found that 17.8% of kindergarten teachers in China earn less than 2000 yuan a month [5]. There are not enough kindergarten teachers to meet the 1:15 student-teacher ratio [28]. Additionally, compared with Western countries, due to larger class sizes, with 31.3% of classes having more than 35 children [5], Chinese kindergarten teachers need to maintain frequent communication with parents [29]. Furthermore, kindergarten teachers in China encounter the challenge of juggling multiple responsibilities, as they are not only responsible for daily instruction but also tasked with submitting various materials, participating in skills competitions, and conducting open classes [30]. Most kindergarten teachers, specifically 92.1%, exceed the standard eight-hour workday [5]. These factors lead to the reduction of Chinese kindergarten teachers’ rest time and their inability to get the salary that matches their efforts, which reduces their occupational well-being and increases their tendency to quit the profession. Findings from a study conducted in China revealed that 38.3% of kindergarten teachers expressed a desire to explore alternative employment options [5]. In some areas, the turnover rate of kindergarten teachers has reached more than 50% [27].

### Person-job fit and occupational commitment

The concept of person-job fit pertains to the degree of congruence between an individual and a job [31]. Parsons developed a trait factor theory on person-job fit grounded on the psychology of individual differences, positing that personality is idiosyncratic, each profession has a distinct personality profile, and individuals are more likely to attain career success by selecting an occupation that aligns with their personality traits [32]. The high matching between occupational interest and occupational demand will produce high occupational commitment, and higher occupational commitment leads to better professional performance [33]. In accordance with career choice theory, the alignment of interests and careers is crucial in the decision-making process, as it results in increased career satisfaction, perseverance, and job performance [34]. The social exchange theory posits that individuals engage in a process of evaluating uncertainties and risks when establishing exchange relations. The outcomes of this risk assessment significantly influence individuals’ attitudes and behaviors towards said exchange relations [35]. According to the social exchange theory, when an individual perceives person-job fit, the result of individual risk assessment is that the benefit is higher than the loss, which will positively affect the attitude and emotion of the individual exchange relationship, such as emotional commitment [35]. Huang, Yuan, and Li discovered that employees who possess adequate knowledge and skills to fulfill their job responsibilities are better equipped to manage the innovation process, exhibit higher levels of work engagement, and demonstrate stronger occupational commitment [36].

Furthermore, the match between knowledge employees and their positions is a crucial determinant of the retention rate [37]. Certain scholars have explicitly emphasized the importance of addressing the matter of person-job fit meticulously to ensure organizational stability and longevity, and assisting decision makers in selecting the most suitable candidates for the job through competency perception matching and personality trait identification [38]. The approach to attaining the alignment between individuals and jobs can be examined from two angles: need-supply (N-S) and demand-ability (D-A) [39]. The former posits that the individual’s needs, desires, and aspirations are met by the job, while the latter emphasizes the individual’s ability to meet the job’s requirements. At present, the salary of preschool teachers in China is generally low, which cannot meet their needs [5]. Moreover, more than 50% of kindergarten teachers have college degrees or below, and the educational level cannot meet the needs of kindergarten education [5]. From the perspective of employees, in order to improve the person-job fit of employees, organizations need to constantly improve the working environment [21]. When the work environment meets the development needs of employees, it fosters their initiative and motivates them to strive to improve their professional competence and align with work standards.

### Occupational well-being as a potential mediator

Occupational well-being, a constructive emotional experience that emerges within the workplace, serves as a viable metric for assessing employees’ emotional experiences and evaluative judgments of their occupations [40]. Improving the occupational well-being of employees is of great significance because occupational well-being is essential to the survival and development of any organization in the world [41]. The occupational well-being of teachers has a significant effect on education, teaching, and research within the school field. The occupational well-being is helpful to construct teachers’ self-identity and promote successful educational reform [19]. Scholarly research suggests that teachers who experience positive emotions and a sense of well-being are better equipped to promote the happiness and development of their students [42, 43]. Occupational well-being has the function of retaining and motivating high-quality employees [44]. Educators with elevated levels of occupational well-being exhibit greater willingness to dedicate their time and effort to teaching and research, as well as a higher inclination to remain within the educational institution [41]. The cultivation of occupational well-being can expand the social and resourceful domains for employee growth, enhance their motivation to contribute to the organization, and elevate productivity, attendance, and retention rates [45]. Occupational well-being is a kind of positive psychological feeling and cognition. Employees with strong occupational well-being get more satisfaction and happiness from work, and the more they love their career, thus forming higher emotional commitment to their career [46]. The occupational well-being of kindergarten teachers is deeply intertwined with their educational responsibilities, and exhibits a positive correlation with their occupational commitment [42]. With the improvement of preschool education in China, all sectors of society have higher and higher requirements for kindergarten teachers, but the level of respect for kindergarten teachers’ profession has not been improved, and the occupational well-being of kindergarten teachers is low [28].

The congruence between an individual and their work environment is a fundamental requirement for promoting employee well-being [47]. Social exchange theory shows that social exchange originates from social attraction [48]. Person-job fit means that the individual can provide knowledge and ability to meet the requirements of the job, and the job can provide remuneration and resources to meet the needs of the individual. In this way, individuals and work can attract each other, and this attraction promotes the exchange of individuals and work. In the context of reciprocal exchange, individuals are inclined to reciprocate positive emotions or work attitudes as they engage in work activities [35]. When individuals align with the organizational values, they are more likely to experience a sense of belonging, identify with the organization, derive meaning from their work, and derive greater happiness from the work process [49]. Moreover, when N-S matching is realized, individuals believe that their efforts are recognized by the organization and rewarded accordingly, and can experience more positive emotions in the work process [39]. On the other hand, D-A matching means that individuals have professional skills, can better adapt to, be competent and complete work tasks, get recognition, praise and reward from the organization, and then experience more positive emotions in work [39]. When person-job fit is realized, individual job satisfaction will increase [50]. Job satisfaction is the core element of the operational definition of occupational well-being [35]. Thus, it can be deduced that person-job fit potentially exerts a positive influence on individual occupational well-being. Conversely, when the attributes of the workplace fail to align with an individual’s skills or preferences, it can result in stress and have a detrimental effect on their occupational well-being [51]. Csikszentmihalyi, a prominent figure in the field of positive psychology, has highlighted the significance of achieving an optimal balance between the difficulty of the work task and an individual’s skills, which can lead to a state of mind-flow characterized by complete engagement in the task at hand [11]. The equilibrium between work effort and reward, as well as between needs and resources, holds particular importance for the promotion of occupational well-being [52].

### Perceived organizational support as a potential moderator

In 1986, Eisenberger and colleagues introduced the notion of Perceived Organizational Support (POS), which pertains to the degree to which employees perceive that their organizations prioritize their welfare and appreciate their contributions [53]. Drawing on the social exchange theory, POS is posited to fulfill employees’ desire for commendation and acknowledgement, thereby fostering a favorable emotional bond with the organization. This emotional connection, in turn, stimulates employees’ exertion and bolsters the attainment of organizational objectives. The provision of organizational support, deemed a crucial environmental resource, serves to mitigate the stress experienced by individuals who struggle to adapt to their work environment [51]. Particularly in the face of demanding work tasks, the perceived organizational support, a significant safeguard against adverse outcomes, can serve as a motivating force that not only facilitates successful task completion, but also augments employee competencies [54]. In the context of educational institutions, the provision of affirmative feedback to peers, receipt of assistance from colleagues, and alignment of pedagogical beliefs among colleagues can yield favorable outcomes for teachers’ professional dedication [16]. However, studies have found that kindergarten teachers in China have less support from their organizations, 38.1% of teachers did not get comfort from colleagues and leaders when they encountered difficulties, and 36.2% of teachers rarely shared happiness and sadness with colleagues and leaders [5].

Additionally, the perceived organizational support serves as a moderator in the relationship between employees’ suitability for their jobs and their level of work engagement [55], which, in turn, is significantly linked to occupational commitment [56]. In their study, Sun and Zhou found that the perceived organizational support among kindergarten teachers functions as a moderator in the relationship between person-job fit and burnout [10]. Similarly, Wei reported a significant negative correlation between burnout and the affective and normative commitment of kindergarten teachers towards their profession [57]. As previously stated, person-job fit and perceived organizational support serve as protective factors for occupational commitment. The protective-protective model, posits that these two protective factors may interact and presents two hypotheses to elucidate their combined effects: the enhancing interaction hypothesis and the antagonistic interaction hypothesis [22]. The enhancing interaction hypothesis posits that the effect of one protective factor on adaptive outcomes may be augmented by the presence of the other protective factor. The antagonistic interaction hypothesis posits that the effect of a protective factor on its adaptive outcome may be diminished by another protective factor. The kindergarten work environment presents uncertainty regarding the interplay between perceived organizational support and person-job fit and their influence on teachers’ occupational commitment. Consequently, this study investigates the moderating function of perceived organizational support in the relationship between person-job fit and occupational commitment, without explicitly proposing the direction of perceived organizational support regulation.

### The present study

**Fig. 1:**
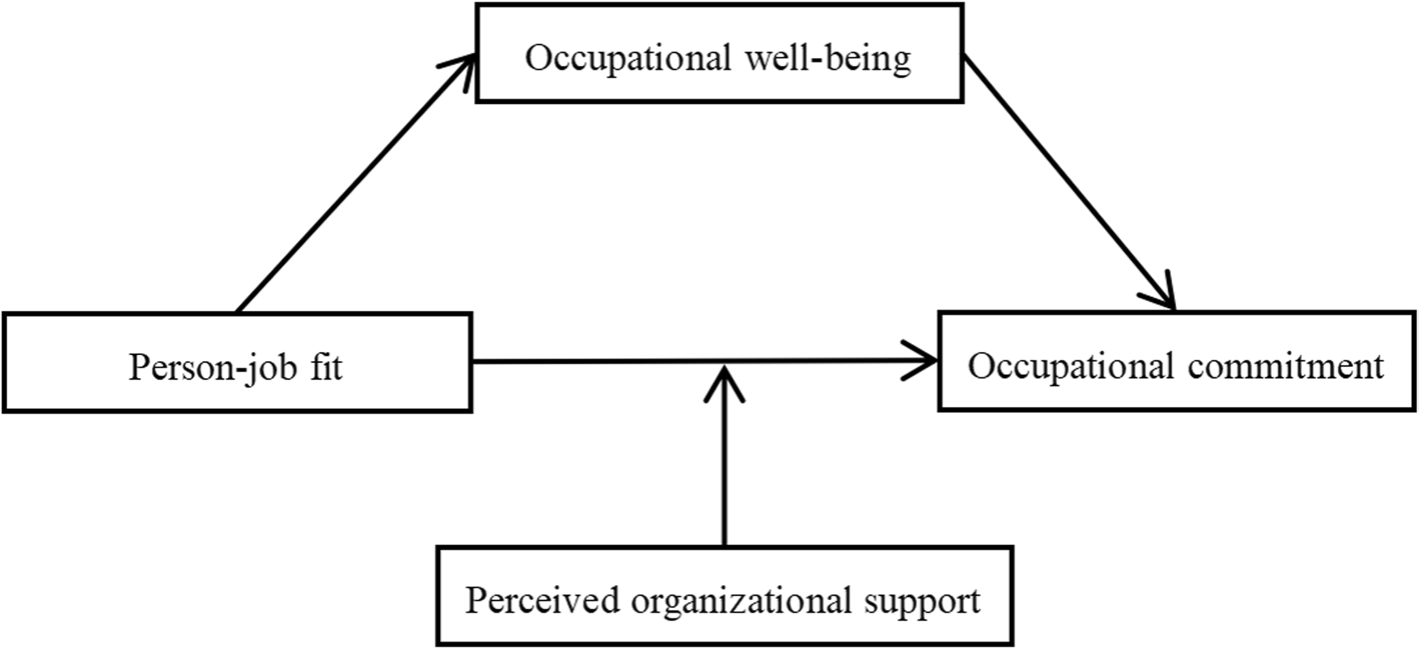
Theoretical hypothesis model diagram of this study

Drawing from the preceding discourse, we have posited three hypotheses (see Fig. 1): (1) There is a significant positive relationship between person-job fit and occupational commitment; (2) Occupational well-being mediates the relationship between person-job fit and occupational commitment; (3) Perceived organizational support moderates the relationship between person-job fit and occupational commitment.

## Method

### Participants and procedure

The Ethics Committee of Guangxi Normal University approved the study (IRB NO. ZS025). The researcher distributed a questionnaire to kindergarten teachers via a WeChat group comprised of preschool education graduates from eight Chinese universities. The researcher provided a comprehensive explanation of the study’s objectives to the graduates and assured them that the results would be utilized solely for research purposes, without compromising the privacy of the teachers, including their names and contact details. Prior to responding to the questionnaire, the participants were requested to peruse the privacy statement of the investigation. A study involving 1561 kindergarten teachers was conducted, with 22 samples exhibiting strong regularity being excluded. Ultimately, data from 1539 teachers were considered valid and included in the analysis, with a valid questionnaire rate of 98.59% (see Table 1).

**Table 1:** Demographics of the participants (*N* = 1539)

Basic Data	Item	Amount (%)
Workplace	Urban	958 (62.25%)
	Non-urban	581 (37.75%)
Nature of the institution	Public schools	992 (64.46%)
	Private schools	547 (35.54%)
Daily working hours	8 hours or less	514 (33.40%)
	8–10 hours	836 (54.32%)
	10–11 hours	157 (10.20%)
	11 hours or more	32 (2.08%)
Gender	Male	402 (26.12%)
	Female	1137(73.88%)
Grade	Junior grade of kindergarten	753 (48.93%)
	Mid-grade of kindergarten	394 (25.60%)
	Senior grade of kindergarten	392 (25.47%)
Education	High school and below	276 (17.93%)
	College	482 (31.32%)
	Bachelor	716 (46.52%)
	Master	65 (4.22%)
Majors	preschool education	1015 (65.95%)
	non-preschool education	524 (34.05%)
Years of teaching experience	0–1 years	410 (26.64%)
	2–5 years	691 (44.90%)
	6–10 years	245 (15.92%)
	11–20 years	122 (7.93%)
	20 + years	71 (4.61%)
Average monthly income	RMB Less than 2000	379(24.62%)
	RMB 2000–3000	527(34.24%)
	RMB 3000–4000	262(17.02%)
	RMB 4000–5000	177(11.50%)
	RMB 5000–10,000	151(9.81%)
	RMB More than 10,000	43 (2.79%)
Age	18–25 years old	650 (42.24%)
	26–35 years old	602 (39.12%)
	36–45 years old	224 (14.55%)
	Age 46 +	63 (4.09%)
Status	Regular employee	1187 (77.13%)
	Temporary employee	352 (22.87%)

### Measures

#### Person-job fit

This study used Wong’s revised Person-Job Fit Scale [58], which comprises four items, one of which is “The work environment offered by my organization matches my job requirements.”The scale is evaluated on a 5-point Likert-type scale, with higher scores indicating a stronger alignment between the individual and the job. Sun and Zhou have confirmed the scale’s validity for use in research with kindergarten teachers [10]. In the current study, the Cronbach’s alpha (α) for this scale was 0.87.

#### Perceived organizational support

The present study used the Perceived Organizational Support Scale revised by Liu et al., which comprises six items, one of which is “The unit will consider my opinion” [59]. The scale is rated on a 5-point Likert-type scale, with higher scores indicating greater levels of perceived organizational support. Previous research has demonstrated the utility of this scale in investigations involving kindergarten teachers [10]. The α for the entire scale in this study was 0.92.

#### Occupational well-being

The Occupational Well-being Scale for Early Childhood Teachers used in this study was revised by Wang [60]. The scale comprises 15 items, one of which is “I feel in control of my work”, including four subscales of psychological, emotional, social, and cognitive well-being. The scale is evaluated on a 3-point Likert-type scale, with elevated scores indicating greater levels of occupational well-being. We used the total score as an index to evaluate the occupational well-being of kindergarten teachers. In this study, the α for this scale was 0.92.

#### Occupational commitment

The Teacher Occupational Commitment Scale used in this study was developed by Long and Li [61]. Comprising 16 items, the scale encompasses three subscales, namely affective, continuance, and normative commitment. The items include statements such as “I feel a responsibility to continue from achieving my career.” The scale is rated on a 4-point Likert-type scale, with higher scores indicating greater levels of occupational commitment. Prior research has demonstrated the utility of this scale in examining the commitment of kindergarten teachers [42]. We used the total score as an index to evaluate the occupational commitment of kindergarten teachers. In the current study, the α for the total scale was 0.91.

#### Analysis

The present study employed SPSS 23.0 and Amoss 24.0 to analyze the data. In the first step, the scoring criteria differed among the four scales utilized in the study. Specifically, the Occupational Well-Being Scale adopted a 3-point scale, the Occupational Commitment Scale employed a 4-point scale, while the Person-Job Fit Scale and the Perceived Organizational Support Scale utilized a 5-point scale. To ensure consistency in the scoring criteria, the researchers utilized SPSS to convert the data from the Occupational Well-Being Scale and the Occupational Commitment Scale into a 5-point scale using the formula “Y = (B-A) * (X-a)/(b-a) + A” [62]. When we need to convert a 3-point scale to a 5-point scale, this formula can be stated as “Y = (5-1) * (X-1)/ (3-1) + 1.”

The second step was to evaluate the α through the utilization of SPSS 23.0. A criterion of α above 0.70 was employed to evaluate the reliability of the measure [63]. We applied average variance extracted (AVE) and composite reliability (CR) to evaluate convergent validity, where the values of AVE above 0.50 and CR above 0.70 indicated good convergent validity [63]. The Harman single factor test was used to evaluate whether there was serious common method bias (CMB) in this study. Additionally, confirmatory factor analysis (CFA) was performed using AMOS 24.0. We used χ^2^/df < 5, CFI > 0.90, TLI > 0.90, and RMSEA < 0.08 as the criteria for measuring the goodness of fit of the theoretical hypothesis model [64].

In the third step, SPSS 23.0 was used for difference testing (t test and f test) and correlation analysis. The outcomes of this analysis indicated that eight demographic variables, namely gender, daily working hours, grade, education, years of teaching experience, average monthly income, status, and age, significantly affected the scores of kindergarten teachers in relation to their occupational commitment, person-job fit, perceived organizational support, and occupational well-being. Consequently, we used these variables as control variables.

Finally, we used Model 5 in PROCESS v3.3, a plug-in for SPSS, to analyze the model,

## Results

###  Common method Bias

We employed Harman single-factor test to evaluate the presence of CMB [65]. The results of the Harman single-factor test indicated that the eigenvalues of 11 factors exceeded 1, and the proportion of variance accounted for by the first factor was 34.49% (below 40%). The fitting indexes (χ^2^/df = 21.80, GFI = 0.50, CFI = 0.57, NFI = 0.56, RMSEA = 0.12) of the single factor Confirmatory factor analysis (CFA) model did not meet the standard of good fitting. So, the common method bias of the study was within the acceptable range.

### Reliability and validity of measurement variables

As shown in Table 2, the AVE of each variable in the four-factor model was above 0.50 and the CR values were all above 0.70. Further, the α for each variable in this study were above 0.70. Hence, this study has good reliability and validity.

**Table 2:** Reliability and convergent validity (*N* = 1539)

	α	AVE	CR
Person-job fit	0.87	0.63	0.87
Perceived organizational support	0.92	0.66	0.92
Occupational well-being	0.92	0.51	0.91
Occupational commitment	0.91	0.52	0.92

### Descriptive statistics and correlations of study variables

As shown in Table 3, there existed a statistically significant positive correlation among the four variables, and the relationship between them need to be further explored.

**Table 3:** Descriptive statistics and correlation analysis (*N* = 1539)

	*M*	*SD*	1	2	3
1. Occupational well-being	32.61	5.34	–		
2. Person-job fit	13.15	3.35	0.45^***^	–	
3. Perceived organizational support	19.61	4.77	0.55^***^	0.56^***^	–
4. Occupational commitment	45.60	6.97	0.54^***^	0.42^***^	0.50^***^

***p < 0.001

### Model analysis

Amoss 24.0 was employed to assess the adequacy of the theoretical hypothesis model (see Fig. 1) proposed in this research. The results showed that χ^2^/df = 4.95, GFI = 0.90, CFI = 0.92, NFI = 0.91, RMSEA = 0.05. This showed that the theoretical assumption model had an acceptable goodness of fit.

**Fig. 2:**
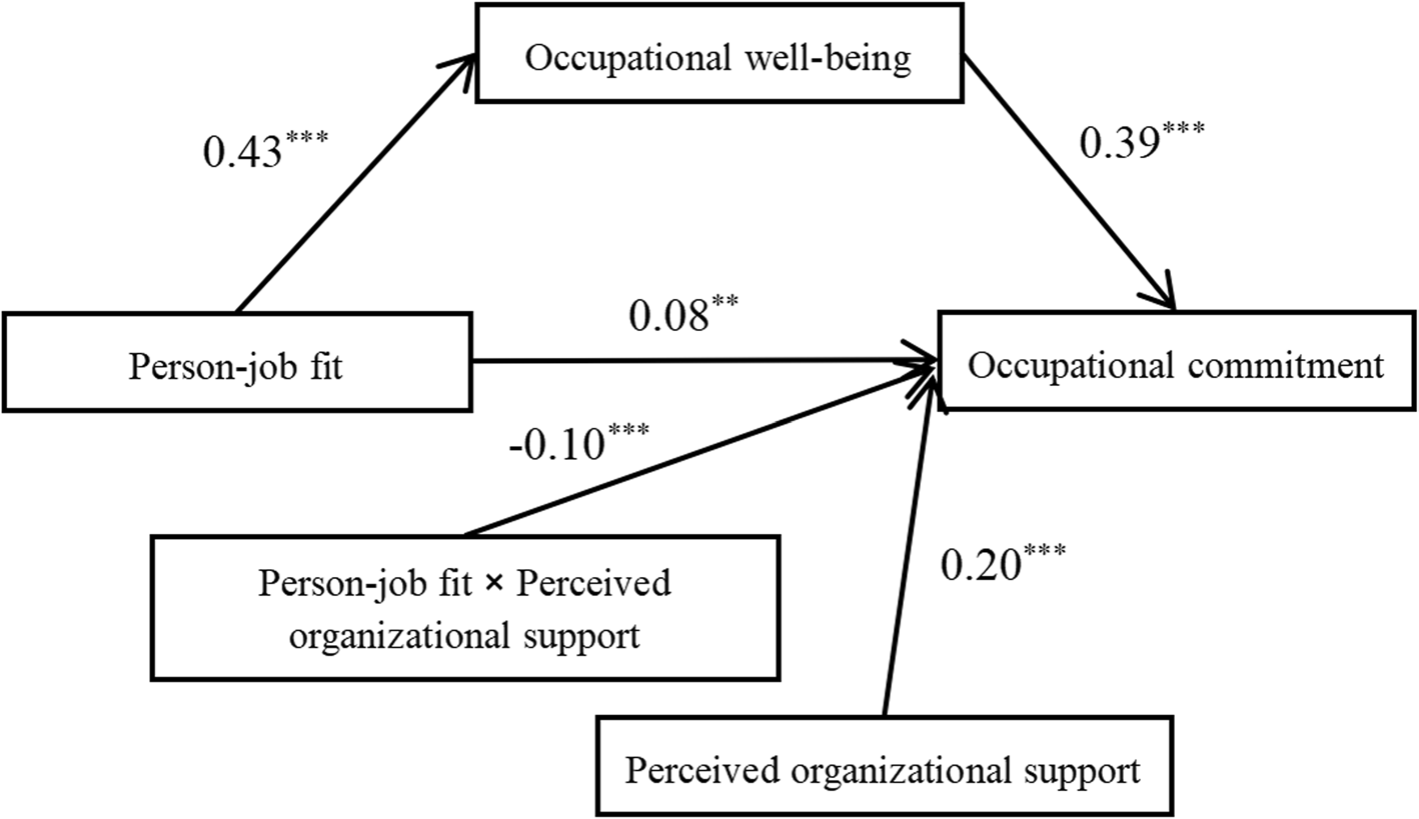
Figure of model analysis results

We analyzed the model using PROCESS 3.3 (Model 5). Control variables included gender, daily working hours, grade, education, years of teaching experience, average monthly income, status, and age. The findings, as presented in Table 4 and Fig. 2, indicate a significant and positive correlation between person-job fit and occupational well-being, as well as a significant and positive correlation between occupational well-being and occupational commitment. Additionally, the direct effect of person-job fit on occupational commitment was found to be significant. As shown in Table 5, the 95% confidence interval of [0.16, 0.24] suggested that occupational well-being served as a mediator for the effect of person-job fit on occupational commitment. As shown in Table 4, the interaction term between person-job fit and perceived organizational support exhibited a significant negative correlation with occupational commitment, indicating that perceived organizational support negatively moderated the association between person-job fit and occupational commitment.

**Table 4:** Model analysis (*N* = 1539)

	Occupational well-being			Occupational commitment			Occupational commitment		
	*β*	*SE*	95% CI	*β*	*SE*	95% CI	*β*	*SE*	95% CI
Person-job fit	0.43^***^	0.02	[0.38, 0.48]	0.21^***^	0.02	[0.16, 0.26]	0.08^**^	0.03	[0.02, 0.13]
Occupational well-being				0.45^***^	0.02	[0.41, 0.50]	0.39^***^	0.02	[0.34, 0.44]
Perceived organizational support							0.20^***^	0.03	[0.15, 0.26]
Person-job fit x perceived organizational support							−0.10^***^	0.02	[−0.13, −0.06]
R^2^	0.24			0.35			0.38		
F	52.46^***^			80.49^***^			78.04^***^		

**p < 0.01, ***p < 0.001; All variables were standardized to ensure comparability

**Table 5:** Decomposition of total effect, direct effect and indirect effect

Effect	Effect value	Boot *SE*	95% CI	Percentage
Total effect	0.41	0.02	[0.36, 0.48]	
Direct effect	0.21	0.03	[0.15, 0.27]	51.22%
Indirect effect	0.20	0.02	[0.16, 0.24]	48.78%

**Fig. 3:**
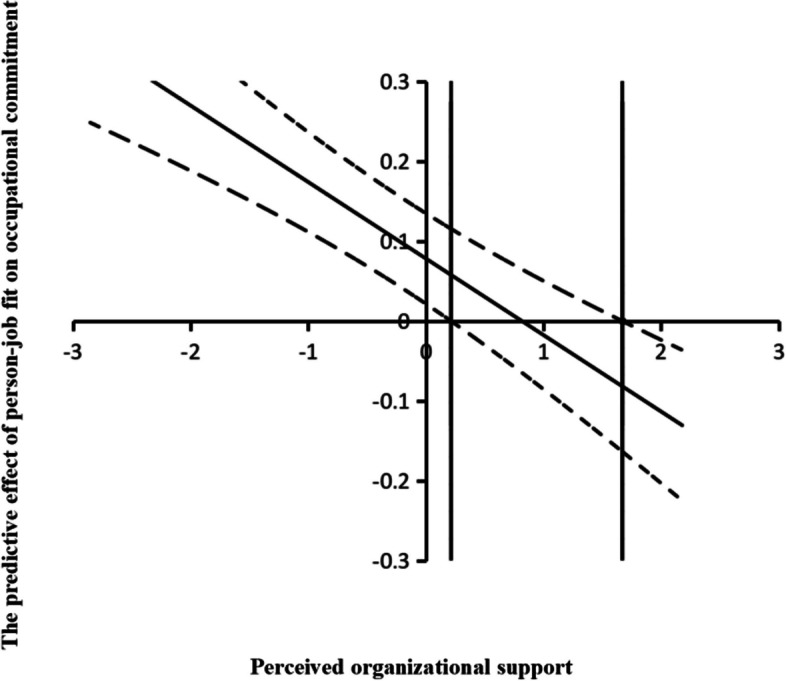
J-N moderating effect of Perceived organizational support. Note. The diagonal solid line represents the regression coefficient of occupational commitment to person-job fit under different perceived organizational support scores. The diagonal dotted line represents the upper and lower bounds of 95% confidence interval of the regression coefficient of occupational commitment to person-job fit under different perceived organizational support scores

To more visually reflect the relationship between person-job fit and occupational commitment when perceived organizational support takes different values, the Johnson-Neyman technique was used to plot the moderating effect (see Fig. 3). As illustrated in Fig. 3, the left vertical line exhibited a value of 0.21, while the right vertical line displayed a value of 1.67. This indicated that a significant positive correlation exists between person-job fit and occupational commitment when the perceived organizational support scores were less than 0.21. By contrast, a statistically significant negative correlation was detected between person-job fit and occupational commitment, with a 95% confidence interval, when the perceived organizational support scores exceeded 1.67.

## Discussion

The present study developed a theoretical model that incorporated occupational well-being as a mediating variable and perceived organizational support as a moderating variable to examine the relationship between person-job fit and occupational commitment among kindergarten teachers. The study findings indicate that: (1) A significant positive correlation exists between person-job fit and occupational commitment; (2) Person-job fit has the potential to augment kindergarten teachers’ occupational commitment by improving their occupational well-being; (3) The relationship between person-job fit and occupational commitment was moderated by perceived organizational support. A significant positive relationship between person-job fit and occupational commitment was observed when perceived organizational support scores were less than 0.21, whereas a significant negative relationship was noted when perceived organizational support scores exceeded 1.67.

### The relationship between person-job fit and occupational commitment

The findings indicate a significant positive relationship between person-job fit and occupational commitment, which aligns with prior empirical research [66–68]. When person-job fit is realized, individual motivation and loyalty will be enhanced [50]. Specifically, when kindergarten teachers’ preferred occupational category corresponds with their current occupational category, they exhibit higher levels of commitment towards their work and demonstrate greater adherence to organizational requirements, which subsequently increases their likelihood of persisting in the profession [66]. According to the social exchange theory, person-job fit means that individuals satisfy job requirements by investing their own knowledge and skills, to obtain resources and support to meet their own development and needs from work, but the knowledge and skills possessed by individuals will not suffer any loss due to the investment in work [35]. Therefore, individuals will have a positive attitude towards the occupation they are engaged in and are willing to continue to engage in the occupation.

Based on the D-A theoretical model [39], kindergarten teachers possessing the requisite knowledge and skills for preschool education are better equipped to perform their duties. Through systematic and professional training, these teachers acquire the necessary expertise to effectively engage in early childhood education, thereby enhancing their qualifications and professional commitment [68], and potentially leading to additional benefits and qualifications [69]. Furthermore, teachers who possess a congruent alignment between their expertise and competencies and their occupational role not only possess adequate comprehension and proficiency to fulfill the job demands, but also exhibit enhanced proficiency in managing the process of innovation [70]. Consequently, kindergarten instructors who exhibit a high degree of person-job fit are likely to experience reduced psychological strain associated with their work, while concurrently increasing their likelihood of receiving both tangible and intangible rewards, and demonstrating a greater propensity to remain in their profession [70]. From a need-supply perspective [39], kindergarten teachers who experience a high degree of person-job fit can satisfy their needs for survival, belonging, and respect through the salary and support offered by their occupation. This, in turn, fosters a more positive attitude towards work and cultivates internal motivation to remain in the profession.

### The mediating role of occupational well-being

The results indicated that occupational well-being serves as a partial mediator in the relationship between person-job fit and occupational commitment, aligning with prior research [71]. According to the social exchange theory, person-job fit, as a feature of work situation, can be perceived by individuals as a resource supply, which can promote individuals to have a positive attitude towards work [72]. N-S model holds that person-job fit means that jobs can meet individual needs or preferences [73]. Additionally, the satisfaction of teachers’ needs is a source of positive affect [74]. Therefore, the higher the degree of person-job fit, the more satisfaction individuals get from work, and the higher their occupational well-being. According to the flow theory of positive psychology [75], when kindergarten teachers engage in work that matches the difficulty and demands of their work with their own personality traits and knowledge and skills, they are motivated to work intrinsically, focus all their attention, devote themselves to their work, and obtain a positive emotional experience from the profession.

In contrast to assembly line laborers, kindergarten teachers are unable to execute monotonous duties within the confines of prescribed guidelines and regulations. They are required to confront numerous unforeseen challenges and must also discern prospects for the advancement of children, while creating inventive curricula through their interactions with them. The Broaden-Build theory posits that occupational well-being, as a positive emotion, can augment the working memory and verbal fluency of kindergarten teachers by broadening their attentional scope and range of action [76]. This, in turn, fosters cognitive flexibility, creativity, and reinforces their professional convictions. Consequently, occupational well-being not only facilitates the realization of professional objectives and self-esteem but also engenders an intrinsic motivation to balance work resources and demands, thereby establishing a robust emotional connection with their occupation [52].

### Moderating effect of perceived organizational support

The findings of current study indicate that the relationship between person-job fit and occupational commitment was moderated by perceived organizational support, aligning with previous research conducted by Sun and Du [68]. Notably, a significant positive relationship between person-job fit and occupational commitment was observed when perceived organizational support scores were below 0.21, while a significant negative relationship was observed when scores exceeded 1.67. The observed outcome can potentially be attributed to the compensatory effect of person-job fit and perceived organizational support, both of which serve as protective factors for occupational commitment. This finding substantiates and broadens the antagonistic interaction hypothesis of the protective-protective model [22], which states that one protective factor may not only reduce the effect of the other protective factor but may even cause the effect to go in the opposite direction. This finding suggests that the interplay among occupational commitment, person-job fit, and perceived organizational support may be subject to various contextual factors, including industry type and cultural background, resulting in a nuanced and intricate relationship that warrants further investigation and refinement through additional research.

The reasons for this result are analyzed at a practical level. The characteristics of preschool children’s physical and mental developmental stages determine the variable and sudden nature of kindergarten teachers’ work, which leads them to work in a process of frequent high emotional labor and in dire need of social support from the work environment, otherwise they are prone to emotional exhaustion [19]. However, the results of the current large-scale survey of kindergarten teachers in China found that kindergarten teachers’ perceived levels of organizational support were low [5]. In situations where kindergarten teachers perceive inadequate organizational support, particularly when their perceived organizational support scores fall below 0.21, the importance of the fit between kindergarten teachers and their positions becomes crucial in elevating their level of occupational commitment.

### Limitations

Despite the ability of the sample size and research design employed in this study to attain the research objectives, there exist four limitations, akin to other studies, which offer directions for future research. The first limitation pertains to the utilization of Chinese kindergarten teachers as the study participants, necessitating further investigation to determine the generalizability of the findings to other groups. Second, the present study employed a self-report methodology for data collection, which may have resulted in measurement error due to social response bias. To address this limitation, future research may benefit from the development of scales that incorporate input from various sources, such as parents, leaders, and colleagues, to investigate the determinants of occupational commitment among kindergarten teachers. Third, as per our findings, occupational well-being served as a partial mediator in the relationship between person-job fit and occupational commitment. Further research could delve into other potential mediating factors, including burnout, perceptions of affiliation, and professional identity, in order to expand upon the current understanding of why kindergarten teachers with greater person-job fit exhibit heightened levels of occupational commitment. Fourthly, it is important to note that our research design does not allow for the determination of causality. However, in future studies, we can elucidate the causal relationship between variables through experimental or longitudinal investigations. For instance, by measuring each variable at various time points, we can gain insight into the developmental process of kindergarten teachers’ occupational commitment, perceived organizational support, occupational well-being, and person-post match across different time periods. Additionally, cross-lagged regression analysis can be employed to clarify the causal relationship among these variables.

### Implications

#### Theoretical implications

First, the results of this study showed that the person-job fit could not only have a direct effect on occupational commitment, but also affect occupational commitment through the mediating role of occupational well-being. This helps researchers to understand the relationship between person-job fit and occupational commitment from different perspectives. Furthermore, the moderating effect of perceived organizational support on the association between person-job fit and occupational commitment provides support for and expands upon the antagonistic interaction hypothesis posited by the protective-protective model [22], which suggests that a protective factor may not only mitigate the effect of another protective factor but may also cause the effect of another protective factor to shift in the opposite direction.

#### Practical implications

The findings of this study can serve as a valuable resource for preschool policymakers and kindergarten administrators seeking to enhance the occupational commitment of kindergarten teachers. Specifically, the study’s results substantiate the positive influence of person-job fit on kindergarten teachers’ occupational commitment. As such, it is recommended that policy support and financial investment be augmented in pre-service training, recruitment, and post-service training to optimize the alignment between kindergarten teachers and their roles [21]. In the pre-service training phase, our focus is on enhancing the awareness of pre-service teachers regarding their responsibility and mission in the field of kindergarten education. In the recruitment process, we can employ a high reliability assessment tool that is grounded in McClelland’s competency model to identify kindergarten teachers who possess a suitable alignment with the organizational culture, values, and job competency requirements of the kindergarten. Upon the arrival of new kindergarten teachers, we can facilitate their acclimation to the organizational culture and vision of the institution, as well as encourage interaction with colleagues, through the implementation of induction training and various group activities. This process fosters mutual understanding and strengthens the compatibility between new teachers and the work environment of the kindergarten.

Second, person-job fit can have a positive effect on occupational commitment by enhancing kindergarten teachers’ occupational well-being. As such, preschool policy makers and kindergarten administrators should be mindful of the importance of developing intervention programs that facilitate positive emotional experiences for kindergarten teachers through job compatibility. This endeavor necessitates a collaborative effort from all sectors of society. To reduce the emphasis on kindergarten teachers’ “care” and “caregiving” functions in policy and public discourse [77], and to build the professional image of kindergarten teachers as educators [19], and allow them to receive salaries and social status that match their professionalism and importance [5]. In this way, kindergarten teachers can receive the recognition and respect they deserve, so that they can deeply experience the happiness stemming from the profession itself, which engenders an enthusiastic commitment to teaching.

Third, the moderating role of perceived organizational support highlights the importance for preschool policy makers and kindergarten administrators to remain cognizant of the unique psychological needs of kindergarten teachers, both individually and collectively, in order to effectively leverage perceived organizational support as a valuable work resource. To achieve this, kindergarten organizations must demonstrate genuine support for their teachers through three key avenues: supporting their work, attending to their interests, and acknowledging their values [78]. Kindergarten institutions ought to provide their teachers with greater educational autonomy, while adhering to strict ethical guidelines, and bolster their subjective motivation. It is imperative to establish a just and rational evaluation criterion and promotion mechanism, enabling each teacher to enhance their competencies and showcase their strengths, while feeling valued as an integral component of the kindergarten organization. As a manager of a kindergarten, it is imperative to cultivate a harmonious, warm, and cordial organizational atmosphere, while also recognizing and valuing the unique contributions of each teacher. Providing positive evaluations, support, and respect to these individuals is essential.

## Conclusion

Person-job fit, occupational well-being, and perceived organizational support were all significant predictors of occupational commitment. Person-job fit can affect occupational commitment not only directly, but also through the mediating effect of occupational well-being. There is a compensatory effect between person-job fit and perceived organizational support.

## Data Availability

In order to facilitate the utilization of these data in future studies, the authors invite other researchers who may be interested or require relevant data to contact the corresponding author via email at psyzhangshy2018@mailbox.gxnu.edu.cn.
